# Blocking CD30 on CD19 CAR T cells augments their functional capacities against B-cell leukemia/lymphoma

**DOI:** 10.3389/fimmu.2026.1725641

**Published:** 2026-04-30

**Authors:** Tim Schlierkamp-Voosen, Markus Barden, Helena Janning, Marcell Kaljanac, Dennis Christoph Harrer, Celine Dreger, Kathrin Hammon, Moritz Ertelt, Clara T. Schoeder, Hólmfridur Rósa Halldórsdóttir, Carlos Rodriguez-Pardo, Sine Reker Hadrup, Simone Thomas, Hinrich Abken

**Affiliations:** 1 Div. Genetic Immunotherapy, Leibniz Institute for Immunotherapy, Regensburg, Germany; 2 Department of Internal Medicine III, University Hospital Regensburg, Regensburg, Germany; 3 Institute for Drug Discovery, Faculty of Medicine, Leipzig University, Leipzig, Germany; 4 Center for Scalable Data Analytics and Artificial Intelligence ScaDS. AI, Dresden/Leipzig, Germany; 5 Department of Health Technology, Technical University of Denmark, Kongens Lyngby, Denmark

**Keywords:** CAR (chimeric antigen receptor), CD19, CD30, leukemia, lymphoma, T cell

## Abstract

Adoptive therapy with CD19 chimeric antigen receptor (CAR) T cells is effective against B-cell malignancies; however, T-cell activation rapidly declines, increasing the risk of early leukemia relapses. To augment CD19 CAR T-cell functionality, we interfered with the physiological control of T-cell activation by a CAR that prevents the T cell’s inherent CD30–CD30L interaction. Redirected by the bispecific CD30/CD19 CAR, T cells were superior to canonical CD19 CAR T cells in eliminating CD30^-^CD19^+^ cells. Also, CD19^+^ B acute leukemia cells from patients were more efficiently eliminated by the CD30/CD19 CAR T cells *in vitro* than by CD19 CAR T cells. For simultaneous CD30 blocking and CD19 targeting, the scFv order CD30/CD19 in the CAR was superior to CD19/CD30 in forming an optimal synaptic gap. The extended T-cell functionality was not due to binding of any two targets since the bispecific CD20/CD19 CAR was less efficient. Preventing the CD30–CD30L interaction results in reduced TRAF2 levels, increased IκBα, late partial feedback Ser529-p65 phosphorylation, a moderate pSTAT3 elevation, enhanced granzyme B/IFN-γ production, and a dampened Th2 polarization, shifting CD8+ CAR T cells from an immunosuppressive to a more cytotoxic phenotype. Our data demonstrate a strategy in augmenting the efficacy of CD19 CAR T cells against B-cell malignancies by concomitant blocking the T cell’s own CD30–CD30L interaction.

## Graphical Abstract

**Figure:**
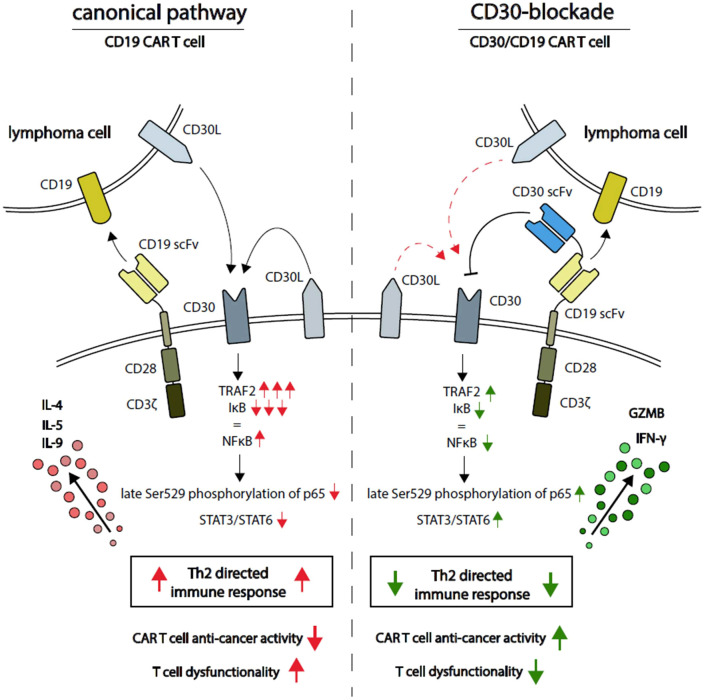
Illustrating the proposed action of the CD30/CD19 CAR on T cells.

## Introduction

CD19 chimeric antigen receptor (CAR) T-cell therapy showed unprecedented efficacy in a variety of B-cell malignancies (1, 2) reaching second-line treatment in primary refractory or early relapsed large B-cell lymphoma (3). However, in third-line treatment, most patients suffer from relapses within months after initial tumor regression. Several reasons account for the decline in therapeutic efficacy, including the gradual loss of T-cell antitumor activity along with the loss of CD19 by leukemia cells. The latter can be addressed by simultaneously targeting two leukemia-associated antigens by bispecific CARs like the CD19/CD20 or CD19/CD22 CAR (4). While efficacious, safe, and reducing the relapse rates in clinical trials (5–7), targeting two antigens on leukemia cells did not prevent from rapid decline in CAR T-cell functional capacities.

Much effort is currently being made to address key regulators that prolong T-cell activation and/or prevent the entry of CAR T cells into a dysfunctional state. Here, we explored a strategy to interfere with a CD19 CAR T-cell inherent mechanism that physiologically limits the pro-inflammatory response. Upon primary activation, T cells upregulate CD30, a tumor necrosis factor (TNF) receptor superfamily member originally identified as a Hodgkin lymphoma antigen (8), to orchestrate the secondary course of T-cell activation and amplification (9). CD30 binding to the CD30 ligand (CD30L) on the same T cell (10) alters T-cell activation by downstream signaling through TNF receptor-associated factor (TRAF) 1, 2, 3, 5 and finally nuclear factor κ-light-chain-enhancer of activated B cells (NFκB) to promote signal transducer and activator of transcription (STAT) 3 and STAT6 phosphorylation and mitogen-activated protein (MAP) kinase activation (11). CD30 activation results in T helper cell (Th) 2-dominated responses that may be associated with further increase in CD30 levels (12, 13) through the interferon regulatory factor 4 (IRF-4)-driven positive feedback loop (14). We recently revealed that secondary T-cell receptor (TCR)/CD28 stimulation of activated T cells in the presence of resting allogeneic or autologous T cells increased the number of CD30^+^ T cells; the process can be counteracted by CD30 CAR T cells blocking the CD30–CD30L interaction (15), indicating the involvement of CD30 in regulating the T-cell immune response.

The T-cell functionality is moreover impacted by a crosstalk between CD30L-expressing malignant cells and CD30 on activated T cells. As an example, CD30L on acute myeloid leukemia (AML) blasts interacts with CD30 on T cells that in turn induces IL-4 secretion (16, 17) and reduces T-cell amplification (18) and their cytotoxic effector functions (19). Both mechanisms, the T-cell intrinsic CD30–CD30L interaction and binding to CD30L on malignant cells, can limit a CAR T-cell response turning an adoptive T-cell therapy inefficient.

We aimed at interfering with the physiological CD30–CD30L-mediated repression of CD19 CAR T-cell activation by blocking CD30 at the CD30L binding site through a CD19 CAR with the additional HRS3 anti-CD30 antibody binding domain. Here, we demonstrated that T cells with the CD30/CD19-specific CAR were superior in eliminating patient CD19^+^ leukemia cells compared with the canonical CD19 CAR T cells. This goes along with dampened Th2 polarization in CAR T cells augmenting and extending the CAR T-cell activation with the objective to finally prevent an early leukemia relapse.

## Methods

### Cells and ethics statement

Peripheral blood mononuclear cells (PBMCs) were isolated from leukocyte reduction system (LRS) cones obtained from blood of healthy donors. Leukemia blasts were isolated from peripheral blood of patients at primary diagnosis by density centrifugation on “Human Pancoll” (PAN-Biotech, Aidenbach, Germany). The studies were conducted in accordance with the Declaration of Helsinki, all donors gave their informed consent, and the protocols were approved by the Ethics Committee of the University Hospital Regensburg (permission numbers 21-2224–101 and 21-2461-101). All patient samples were collected prospectively and routinely underwent a double pseudonymization procedure. T cells were maintained in RPMI 1640 medium supplemented with 4 mM GlutaMAX (Gibco, Thermo Fisher, Waltham, MA, USA), 100 IU/mL penicillin, 100 µg/mL streptomycin (PAN-Biotech), 2 mM HEPES (PAA, GE Healthcare, Freiburg, Germany), and 10% (v/v) heat-inactivated fetal calf serum (PAN-Biotech). HEK293 cells are human embryonic kidney cells (CRL-1573, American Type Culture Collection (ATCC), Manassas, VA, USA); HEK293T cells additionally express the SV40 large T antigen (CRL-3216, ATCC). Nalm6 (CRL-3273, ATCC) is a human acute lymphoblastic leukemia cell line. Cell lines were purchased from ATCC and cultured according to recommended protocols.

### CAR T cells

The CD19-specific (FMC63 scFv) CAR (20) and CD30-specific (HRS3 scFv) CAR (21) with the CD28–CD3ζ signaling domain were previously reported. All CARs are mutated in the lck binding site of CD28 as described (22). The CD30 CAR harbors the HRS3 scFv domain derived from the CD30-specific HRS3 antibody with the capacity to block the CD30–C30L interaction (23). The bispecific CARs in the order CD30/CD19 scFv and CD19/CD30 scFv were engineered by joining the respective scFvs through a (Gly_4_-Ser)_8_ linker. The CEA-specific (BW431 scFv) CAR (21) and CD20/CD19 (2H7 scFv/FMC63 scFv) CAR (20) were used for control. For CAR T-cell manufacturing, T cells were stimulated with the anti-CD3 monoclonal antibody (mAb) OKT-3 (BioLegend, San Diego, CA, USA), CD28 mAb 15E8 (BioLegend), and recombinant human IL-2 (1,000 IU/mL) (Novartis, Basel, Switzerland). IL-2 (200 IU/mL) was added on days 2, 3, and 4 after activation. Retroviral transduction was performed as previously described (24). For enrichment, CAR T cells were labeled with a biotinylated anti-human goat F(ab′)2 IgG antibody (Southern Biotech, Birmingham, AL, USA) followed by purification with anti-biotin microbeads (Miltenyi Biotec, Bergisch Gladbach, Germany). Cells were cultured in presence of IL-2 (200 IU/mL) for 1 week, followed by a 24-h culture period in IL-2 free medium before use.

### Flow cytometry and antibodies

Flow cytometry was performed on BD FACSLyric and BD FACSFortessa and analyzed by FlowJo software version 10.7.1 Express 5 (BD Biosciences, Franklin Lakes, NJ, USA). Cells were incubated with antibodies at 4 °C for 15 min for surface staining. For intracellular staining, cells were fixed and permeabilized with Transcription Buffer or with the Cytofix/Cytoperm Fixation/Permeabilization Kit (BD Biosciences) for 30 min at 4 °C or Transcription Factor Phospho Buffer Set (BD Biosciences) according to the manufacturer’s instructions. The Viability Dye eFluor 780 (Thermo Fisher, Waltham, MA, USA) was employed for live/dead discrimination. Fluorescent-minus-one (FMO) controls were used for gating. For CAR detection fluorochrome-labeled goat anti-human F(ab′)2 antibody (Southern Biotech) was used. The following fluorochrome-labeled mAb were used: anti-CD3 (OKT3 and UCHT1), anti-CD4 (RPA-T4), anti-CD19 (SJ25C1), anti-CD20 (2H7), anti-IRF-4 (IRF-4.3E4) (all BioLegend), Phosflow™ anti-STAT3 (pY705), BD Phosflow™ anti-NF-κB p65 (pS529), anti-human IFN-γ (B27), anti-CD30 (BerH8) and anti-CD8 (RPA-T8) (BD Bioscience), Annexin V (BD Pharmingen), anti-granzyme B (REA226/GB11; Miltenyi Biotec), and anti-PI-9 (2H9D2; Proteintech, Rosemont, IL, USA).

### Cytokine secretion

HEK293 cells were engineered with pQCXIN CMV promoter-driven expression plasmids coding for CD19 or CD30 and used as target cells for CAR T cells. ELISpot assay was performed after 20 h incubation, respectively, as described (25). IL-9 ELISA (R&D Systems, Minneapolis, Minnesota, MN, USA) was performed after 48 h. Th1/Th2 cytokine profile was determined by Human Th1/Th2 Luminex^®^ Performance Assay 13-plex Fixed Panel following the manufacturer’s instructions using MAGPIX (R&D Systems) and analyzed by Belysa Immunoassay Curve Fitting Software, Version 1.0.19 (Sigma-Aldrich, St. Louis, Missouri, MO, USA) after 48 h of coculture.

### Western blot

Immunoblots were performed using the following antibodies: anti-IkBα (44D4), anti-TRAF2 (C192), and anti-α-Tubulin (11H10) (Cell Signaling Technology, Danvers, Massachusetts, MA, USA).

### Cytotoxicity assays

The XTT-based colorimetric assay employing the “Cell Proliferation Kit II” (Roche Diagnostics, Mannheim, Germany) was used to determine the specific viability of target cells after 48 h of coculture with CAR T cells as described (15). For IncuCyte real-time imaging, enhanced green fluorescence protein (eGFP)-labeled Nalm6 cells were used as targets. CAR T cells were cocultivated for 48 h in 96-well flat bottom plates with eGFP Nalm6 cells, microscopic images recorded every 2 h, and the living eGFP^+^ cells quantitatively assessed and normalized to the initial cell number. Analysis of recorded images was performed using the IncuCyte Software 2021C. For recording killing of primary leukemic blasts the “IncuCyte Cytotox Red Dye” (Sartorius, Göttingen, Germany) was added (250 nM).

### Structural modeling of protein–protein interactions

The interactions between the target antigens and extracellular CAR moieties were predicted using AlphaFold2 (26). Distances were measured with the PyRosetta software suite for molecular modeling and design (27), with maximal distances assuming an extended conformation of the linker regions. The predicted structures were visualized using ChimeraX (28) and Created in BioRender. Abken, H. (2026) https://BioRender.com/if16qx5.

### Anti-leukemia activity *in vivo*

For *in vivo* studies, 6-week-old female CIEA NOG immunodeficient mice (NOD.Cg-Prkdcscid-IL2rgTm1sug/JigTac) were acquired from Taconic Labs and housed at the Bio Facility, Department of Health Technology, Technical University of Denmark. All procedures were approved by the Danish National Animal Experiment Inspectorate and the institutional ethical board. 1 × 10^6^ Jeko-1 lymphoma cells expressing luciferase were injected intravenously into 7-week-old NOG mice. Tumors were allowed to engraft for 7 days before randomization and treatment start. On treatment day, 1 × 10^6^ non-modified, CD19 CAR or CD30/C19 CAR T cells were administered i.v. by tail vein injection. Tumor growth was monitored twice per week with bioluminescence imaging (Optical Imaging unit, MILabs). Mice received 30 mg/mL of D-Luciferin intraperitoneally, and bioluminescence was measured 20 min later. Measurements were analyzed using fixed-size regions of interest using MILabs optical imaging software. Mouse body weight and appearance were monitored throughout the experiment.

### Statistical analysis

Statistical analysis was performed using GraphPad Prism, Version 9 (GraphPad Software, San Diego, CA, USA). P values were calculated by Student’s t test, paired t test, or ANOVA as indicated for each data set; ns, not significant; *p ≤ 0.05; **p ≤ 0.01; ***p ≤ 0.001.

## Results

### CD30 blockade improves killing of CD19^+^ target cells

**Figure 1:**
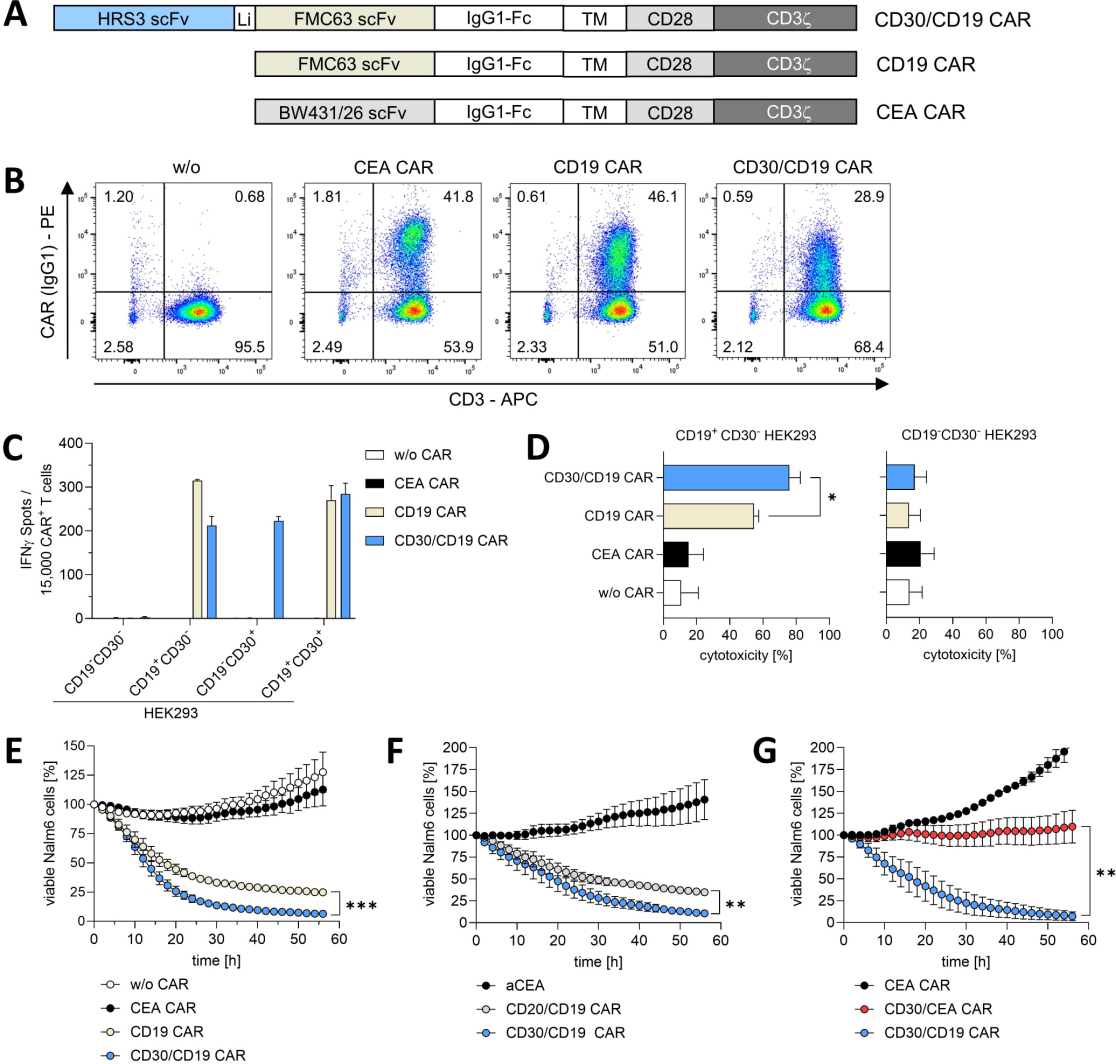
T cells with CD30/CD19 CAR induce superior killing of CD19^+^ target cells. **(A)** Modular composition of the chimeric antigen receptors (CARs). The anti-CD30 single-chain variable fragment (scFv) is derived from the HRS3 antibody, the anti-CD19 scFv from the FMC63 antibody, and the anti-CEA scFv from the BW431/26 antibody which was used as irrelevant specificity control. Li, (Gly_4_-Ser)_8_ linker. **(B)** T cells were engineered by retroviral gene transfer with the respective CAR and recorded by flow cytometry for CD3 and CAR surface expression by the PE-conjugated anti-IgG1 antibody that detects the common IgG1 CH2CH3 spacer within the CAR exodomain. Representative dot plots from one out of three blood donors are shown. **(C)** CAR T cells (5 × 10^4^ CAR^+^ T cells/well) and non-engineered T cells (w/o CAR) were co-incubated with HEK293 cells expressing CD19, CD30, or both at a effector-to-target cell (E:T) ratio of 0.3:1 and monitored for IFN-γ spot formation after 20 h. Data represent mean values of two technical replicates + SEM. **(D)** CAR T cells (2 × 10^4^ CAR^+^ T cells/well) or non-modified T cells (2 × 10^4^ T cells/well) were co-incubated with CD19^+^CD30^-^ or CD19^-^CD30^-^ HEK293 cells (2 × 10^4^ cells/well) for 48 h. Cytotoxicity was determined by an XTT-based viability assay. Data represent mean values pooled from three individual T-cell donors + SEM. Student’s t test: p = 0.0395, Cohen’s D: 2.46, effect size r: 0.776 **(E–G)** Enhanced green fluorescent protein (eGFP)-marked Nalm6 cells (2 × 10^4^ cells/well) were co-incubated with the indicated CAR T cells (2 × 10^4^ cells/well) and cytolysis of Nalm6 leukemia cells was recorded by IncuCyte real-time imaging; cell numbers were normalized to the starting cell number (100%). Data represent mean values pooled from three individual T-cell donors ± SEM. Statistical analysis was performed using the Student’s t test for the 48-h timepoint: **(E)** p = 0.0003; **(F)** p = 0.0055; **(G)** p = 0.0055. *p ≤ 0.05; **p ≤ 0.01; ***p ≤ 0.001.

We aimed at augmenting the functional capacities of canonical CD19 CAR T cells through blocking the intrinsic CD30–CD30L interaction. We therefore engineered the bispecific CD30/CD19 CAR that harbors the CD30 scFv antibody HRS3 for interfering with the CD30–CD30L interaction on the CAR T cell, and the FMC63 scFv for targeting CD19 on the leukemia cell. The CD30 and CD19 scFv binding domains were linked together and grafted on a canonical CAR backbone with the CD28-CD3ζ signaling chain (**Figure 1A**). After retroviral transduction, the CD30/CD19-bispecific CAR was expressed on the T-cell surface, albeit at lower levels than the canonical CD19 CAR (median fluorescence intensity, MFI: 367 vs. 676) (**Figure 1B**).

The CD30/CD19-bispecific CAR activated T cells in an antigen-specific fashion upon encountering HEK293 cells, engineered with CD19, CD30, or both (**Supplementary Figure 1A**), as indicated by the increase of target-specific interferon-γ (IFN-γ) secretion (**Figure 1C**). CAR-driven T-cell activation was antigen-specific since CD19^-^CD30^-^ HEK293 cells did not activate CD30/CD19 CAR T cells, and T cells with a CEA CAR of irrelevant specificity were not activated. Monitoring the cytolytic capacity, CD19^+^CD30^-^ HEK293 cells were lysed by both CD19 CAR T cells and by CD30/CD19 CAR T cells. The lytic efficacy of CD30/CD19 CAR T cells was superior to T cells with the canonical CD19 CAR (**Figure 1D**; p = 0.0395). Cytolysis was specifically driven by the CAR since CEA CAR T cells of irrelevant specificity did not eliminate CD19^+^ HEK293 cells and CD19^-^ HEK293 cells were not lysed by CD19 or CD30/CD19 CAR T cells. Basically, the same results were obtained when using CD19^+^CD20^low^CD30^low^ Nalm6 leukemia cells as targets (**Figure 1E**; **Supplementary Figure 1B**; corresponding Incucyte images **Supplementary Figure 2**). T cells with the CD30/CD19 CAR more efficiently eliminated Nalm6 cells than CD19 CAR T cells in a CD19-specific manner. T cells with the CEA CAR or T cells without CAR did not eliminate Nalm6 cells. Taken together, the data indicated that T cells with the CD30/CD19 CAR were superior in eliminating CD19^+^ cancer cells compared with the canonical CD19 CAR T cells.

We asked whether particularly the CD30 scFv within the bispecific CD30/CD19 CAR format favors enforced T-cell killing of CD19^+^CD20^low^CD30^low^ Nalm6 leukemia cells. We therefore replaced the CD30 scFv by a CD20-specific scFv within the same CAR architecture. As summarized in **Figure 1E**, T cells with the CD30/CD19 CAR showed superior killing of Nalm6 cells compared with CD20/CD19 CAR T cells, indicating that the augmented killing capacity is specifically due to the CD30 scFv domain. Conversely, CD19 is required for recognizing Nalm6 cells since replacing the CD19 scFv by a CEA-specific scFv of irrelevant specificity in the CD30 CAR did not induce substantial Nalm6 cell killing (**Figure 1F**). Data indicate that the superiority of the CD30/CD19 CAR over the canonical CD19 CAR is due to the CD30-targeting domain and not due to the bispecific CAR format itself.

### The distal position of the CD30 scFv is mandatory for improving CAR redirected cytolytic activities

**Figure 2:**
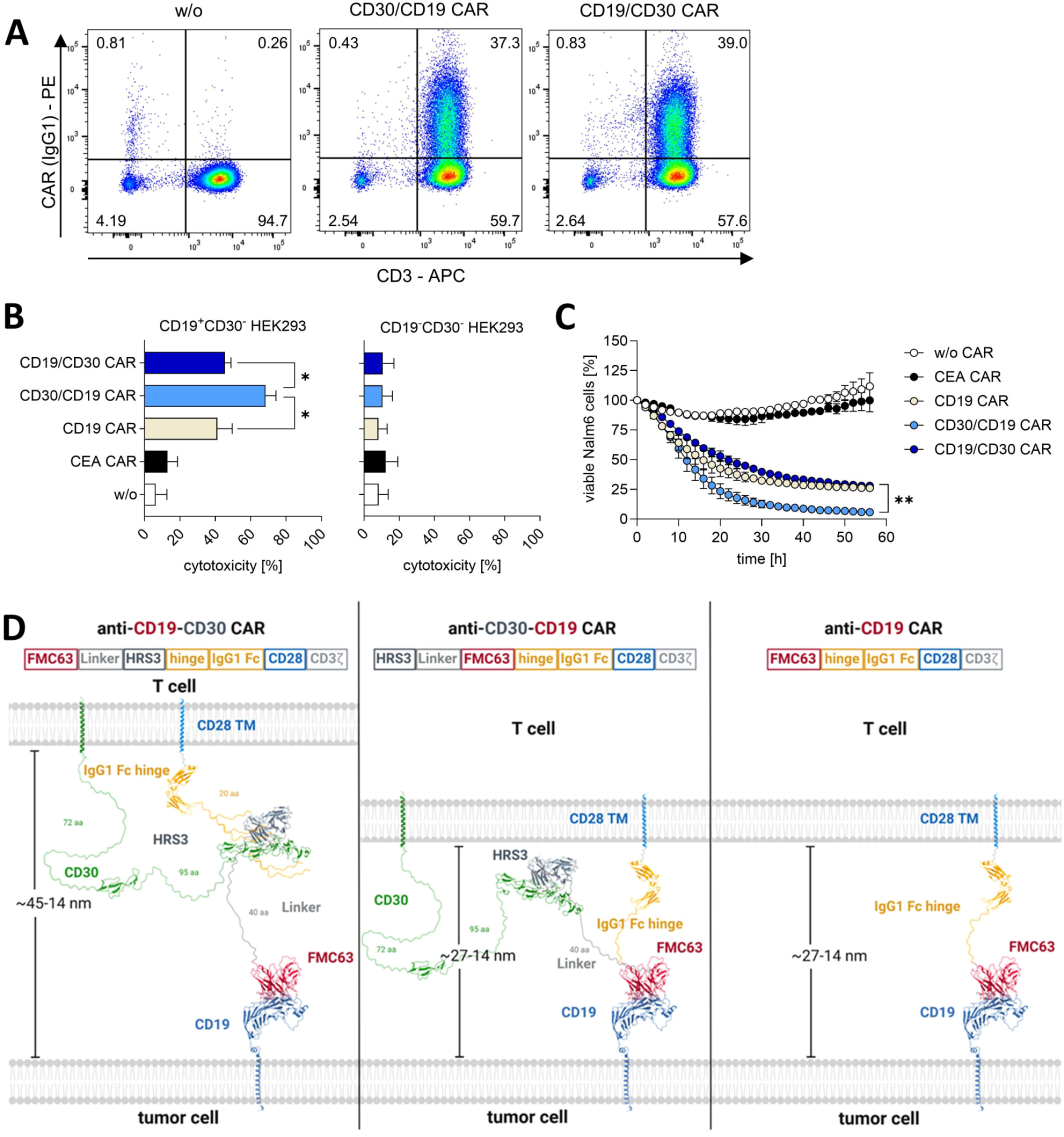
Distal CD30 scFv positioning is essential for enhancing CAR T cell cytolytic activity. **(A)** T cells engineered with the CD30/CD19 CAR and CD19/CD30 were recorded by flow cytometry using the PE-conjugated anti-IgG antibody that detects the common IgG1 CH2CH3 spacer within the exodomain. Representative dot plots from one out of three blood donors are shown. **(B)** T cells engineered with the indicated CAR or non-modified T cells (w/o CAR) (2 × 10^4^ CAR^+^ T cells/well) were co-incubated with CD19^+^CD30^-^ or CD19^-^CD30^-^ HEK293 cells (2 × 10^4^ cells/well) for 48 h. Cytotoxicity was determined by an XTT-based viability assay. Data represent mean values pooled from five individual T-cell donors + SEM. Statistical analysis was performed by one-way ANOVA with Dunnett’s *post-hoc* test: CD19/CD30 CAR vs. CD30/19 CAR: p = 0.042; CD30/19 CAR vs. CD19 CAR: p = 0.0173. **(C)** EGFP^+^ Nalm6 cells (2 × 10^4^ cells) were co-incubated with CAR^+^ or non-modified T cells (2 × 10^4^ cells), and cytolysis of Nalm6 leukemia cells was recorded by IncuCyte real-time imaging. Cell numbers were normalized to the starting cell number (100%). Data represent mean values pooled from two individual T-cell donors ± SEM. Statistical analysis was performed using the Student’s t test for the 48-h timepoint: p = 0.0031. **(D)** Prediction of protein–protein interactions reveals favored synapse formation by the CD30/CD19 CAR. The protein folding of the extracellular moiety of the CD19 CAR, CD30/CD19 CAR, and CD19/CD30 CAR and the most likely mode of interaction with the respective cognate targets CD30 on the T cell and CD19 on the target cell were predicted using AlphaFold2. The predicted synaptic gap between T and target cells was 14–27 nm in the case of the CD30/CD19 CAR and 14–45 nm for the CD19/CD30 CAR. *p ≤ 0.05; **p ≤ 0.01.

The order of the scFvs in the bispecific CAR format may impact target cell recognition and efficiency in eliminating CD19^+^ cells. We addressed this issue by engineering T cells with the CD30/CD19 or the CD19/CD30 CAR and co-incubated the CAR T cells with CD19^+^ HEK293 cells. Both CARs were expressed at the same levels on the T-cell surface (MFI 479 vs. 476) (**Figure 2A**). The cytolytic activity of CD30/CD19 CAR T cells was superior to that of CD19/CD30 CAR T cells; the latter cells were equally efficacious in killing as CD19 CAR T cells (**Figure 2B**). Killing was specifically driven by CD19 recognition since T cells with the CEA-specific CAR and T cells without CAR did not eliminate CD19^+^ HEK293 cells. On the other hand, none of the CAR T cells induced lysis of non-modified HEK293 cells. The same results were obtained upon analyzing the cytotoxicity of CAR T cells toward Nalm6 leukemia cells (**Figure 2C**; p = 0.0031). T cells with the CD30/CD19 CAR produced superior killing of Nalm6 cells compared with CD19/CD30 CAR T cells; the latter CAR T cells were again as efficient as the canonical CD19 CAR T cells. We concluded that the CD30 scFv needs to be at the distal position of the bispecific CAR to augment elimination of CD19^+^ target cells.

To substantiate our conclusion, we conducted structure-based predictions of the CAR exodomain interactions toward CD30 on the T cell and CD19 on the target cell. As illustrated in **Figure 2D**, basically both the CD30/CD19 and CD19/CD30 CAR can interact simultaneously with CD19 and CD30. However, the targeted CD19 epitope is membrane proximal whereas the CD30 epitope is in a membrane distal position of the respective molecule. Therefore, binding by the CD30/CD19 CAR forced a rigid and close membrane-to-membrane convergence leaving a 14–27-nm gap; this is the optimal distance known for major histocompatibility complex (MHC)/TCR-mediated T-cell activation. In contrast, the CD19/CD30 CAR, while bound to its targets, is still more flexible and allows for a gap of 14–45 nm between the T cell and the target cell. In comparison, the canonical CD19 CAR also forced a 14–27-nm membrane gap in the optimal range as did the CD30/CD19 CAR (**Figure 2D**). Overall, the protein–protein interaction models predicted that the CD30/CD19 CAR may form a tighter synaptic gap that is recognized to favor a more stable synapse, in line with the more efficient T-cell activation compared with the CD19/CD30 CAR.

### CD30/CD19 CAR T cells decrease CD30 levels on T cells without inducing fratricide

**Figure 3:**
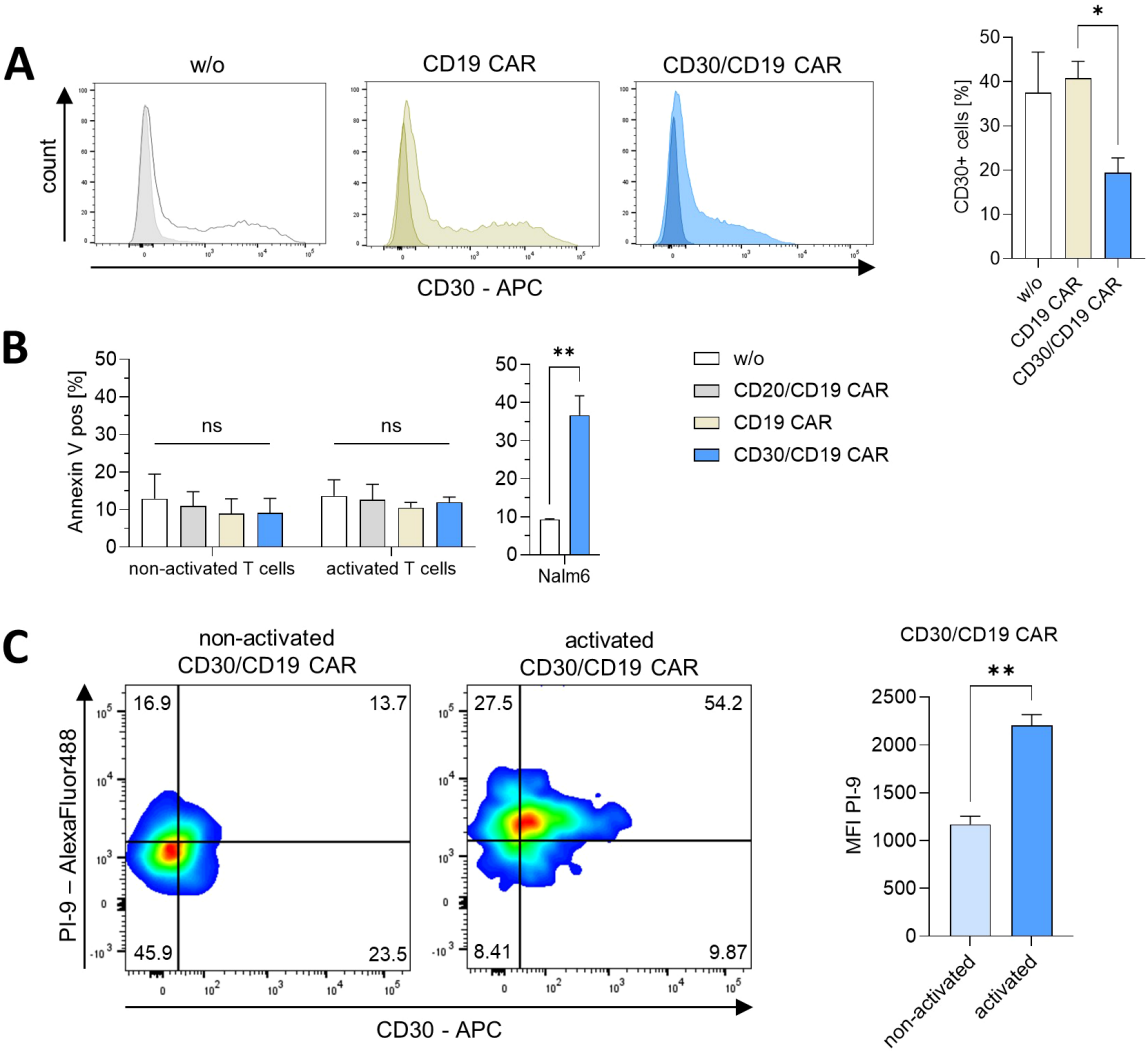
CD30/CD19 CAR T cells downregulate CD30 on T cells without fratricide. **(A)** T cells engineered with the CD19 CAR or the CD30/CD19 CAR (2.5 × 10^4^ cells/well each) were co-incubated together with T cells (w/o CAR) (1 × 10^5^ cells/well) and stimulated with anti-CD3 (OKT3) and anti-CD28 (15E8) antibodies in the presence of IL-2 (1,000 U/mL) for 48 h. CD30 surface expression before (dark-filled histograms) and after stimulation (light-filled histograms) was recorded by flow cytometry. Representative histograms from one out of three T-cell donors are shown. The graph represents mean values of CD30^+^ cells pooled from three individual donors + SEM. Statistical analysis was performed by the Student’s t test: p = 0.0132. **(B)** Non-activated and anti-CD3/anti-CD28-activated T cells (5 × 10^4^ cells/well) were stained with the membrane dye eF450 and co-incubated with CAR T cells (2 × 10^4^ cells/well each) for 6 h. Annexin V-PE staining of membrane-labeled T cells was recorded by flow cytometry. Data represent the mean proportion (%) of Annexin V^+^ cells from three T-cell donors + SEM. Statistical analysis was performed by one-way ANOVA with Dunnett’s *post-hoc* test. p < 0.05 was considered significant (ns, not significant). Nalm6 cells (5 × 10^4^ cells/well) were co-incubated with CD30/CD19 CAR T cells (2 × 10^4^ cells/well) for control demonstrating the killing capacity of the used CAR T cells. Data represent the proportion (%) of Annexin V^+^ Nalm6 cells. Statistical analysis was performed by the Student’s t test: p = 0.0056. **(C)** CD30/CD19 CAR T cells were stimulated through anti-CD3 (OKT3) and anti-CD28 (15E8) antibodies in the presence of IL-2 (1,000 U/mL) for 24 h. CD30 surface expression and PI-9 intracellular expression were monitored by flow cytometry before and after activation. Representative dot plots from one out of three blood donors are shown. Graph represents PI-9 MFI mean values + SEM of sorted CAR T cells pooled from three T-cell donors. Statistical analysis was performed by the Student’s t test: p = 0.0018. *p ≤ 0.05; **p ≤ 0.01.

Activation of healthy donor T cells through TCR/CD3 and CD28 in the presence of IL-2 induces CD30^+^ T cells (15). The same activation regime is routinely used for engineering CAR T cells by retroviral or lentiviral transduction. We therefore asked whether cytokine-induced CD30^+^ T cells are eliminated by CD30/CD19 CAR T cells during or early after manufacturing. T-cell stimulation through TCR/CD28 plus IL-2 induced CD30 expression in a substantial proportion of cells (**Figure 3A**; p = 0.0132). Cocultures of those pre-stimulated T cells with autologous CD19 CAR T cells did not substantially alter the proportion of CD30^+^ T cells. In contrast, the proportion of CD30^+^ T cells, particularly of cells with CD30^high^ levels, was substantially reduced in cocultures containing CD30/CD19 CAR T cells compared with coincubation with CD19 CAR T cells. The observation was not due to antigen masking since we used an antibody for CD30 staining that targets a different CD30 epitope than the HRS3 scFv in the CD30/CD19 CAR.

To address whether fratricide accounts for the reduced number of CD30^+^ T cells, we labeled CD30^-^ non-activated and CD30^+^ activated T cells and co-incubated those cells with CD30/CD19 CAR or CD19 CAR T cells. CD30/CD19 CAR T cells did not increase the proportion of Annexin V^+^ T cells compared with those in the presence of CD19 CAR or CD20/CD19 CAR T cells, or T cells without CAR (**Figure 3B**). We concluded that no substantial fratricide took place. For comparison and as positive control for the killing capacity, CD30/CD19 CAR T cells increased the proportion of Annexin V^+^ Nalm6 cells. We assumed that the CD30/CD19 CAR induced internalization of CD30 from surface upon binding, however, without executing fratricide.

Killing by CAR T cells is predominantly mediated by granzyme B/perforin, which can be prevented by the cell-intrinsic serpin proteinase inhibitor 9 (PI-9) by cleaving granzyme B. Recording the PI-9 levels revealed that T-cell activation through TCR/IL-2 induced a substantial increase in PI-9 levels in the entire T-cell population, also in CD30/CD19 CAR T cells (**Figure 3C**). We hypothesized that these activated T cells are protected from CAR T-cell-mediated fratricide by induced increase in PI-9 levels.

### CD30 blockade dampens Th2 polarization and enhances CAR T-cell cytotoxic function

**Figure 4:**
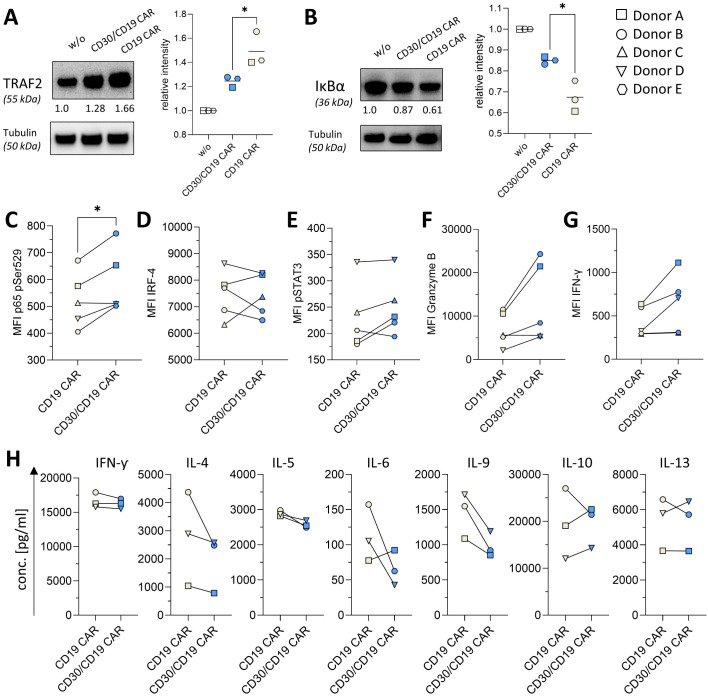
CD30/CD19 CAR T-cell downstream effects of CD30 blockade. **(A, B)** Lysates from T cells engineered with indicated CAR, and from T cells without (w/o) CAR (5 × 10^5^ cells/well each) were prepared after 1 h of co-incubation with CD19^-^CD30^-^ HEK293 cells or CD19^+^CD30^-^ HEK (10 × 10^5^ cells/well each). **(A)** Left panel: representative immunoblot for TRAF2, right panel: relative quantification of protein expression normalized to α-tubulin. **(B)** Left panel: representative immunoblot for IκBα, right panel: relative quantification of protein expression normalized to α-tubulin. The graphs show values from three individual donors. Statistical analysis was performed by the Student’s t test: **(A)** p = 0.0469; **(B)** p = 0.0161. **(C)** p65 (pSer529), **(D)** IRF-4, and **(E)** pSTAT3 protein levels in T cells engineered with indicated CAR were measured by intracellular flow cytometry FACS after 16 h of coculture with CD19^+^CD30^-^ HEK. Data represent MFI values for CD8+ CAR T cells from five individual T cell donors. **(F)** Granzyme B and **(G)** IFN-γ levels in T cells engineered with indicated CAR (5 × 10^4^ cells/well each) were measured by intracellular flow cytometry FACS after 6 h of coculture with CD19^+^CD30^-^ HEK (10 × 10^4^ cells/well each). Data represent MFI values from five individual T-cell donors. Statistical analysis was performed by the Student’s t test: **(C)** p = 0.0254 **(H)** Supernatants of T cells engineered with indicated CAR (5 × 10^4^ cells/well each) after co-cultivation with CD19^+^CD30^-^ HEK (10 × 10^4^ cells/well each) for 48 h were examined for the presence of 11 different target analytes associated with Th1/Th2 immune response (seven analytes displayed, additional analytes in **Supplementary Figure 4A**). Luminex data represent mean values of technical triplicates of three individual T-cell donors. *p ≤ 0.05.

CD30 signaling physiologically recruits TRAF2 to its cytoplasmic domain, activating the canonical NF-κB pathway via IKKβ-mediated IκBα degradation and p50p65 nuclear translocation (29), which promotes Th2-like T-cell differentiation and cytokine secretion patterns that limit effector cytotoxicity (30). Initially, we therefore asked for the TRAF2 and IκBα levels in CAR expressing T cells. T cells engineered with the CD30/CD19 CAR or the CD19 CAR were co-incubated with CD19^+^CD30^-^ HEK293, non-transduced T cells indicating the basic TRAF2 and IκBα levels in the respective T cells as assessed by Western blot after 1 h of stimulation (**Figures 4A, B**). CD30/CD19 CAR T cells showed reduced TRAF2 levels compared with CD19 CAR T cells, whereas IκBα was higher in CD30/CD19 CAR T cells.

Apart from canonical IκBα suppression, our data revealed an increased p65 Ser529 phosphorylation 16 h post-stimulation (**Figure 4C**). Differential p65 phosphorylation acts as a code directing NF-κB transcriptional activity to specific gene subsets, as confirmed by phospho-mutant studies showing site-specific effects on p65/p-RNAP II promoter recruitment and κB enhancer-dependent gene expression (31). Unlike Ser536 (translocation/DNA binding) (32), Ser529 reports transcriptional activation of NF-κB targets post nuclear entry (33, 34); a late S529 increase despite early IκBα increase may indicate partial feedback transcription.

CD30 ligation activates the non-canonical NF-κB activation (through p52/RelB dimers), which transcriptionally induces IRF-4 expression; IRF-4 reciprocally upregulates CD30, forming a positive feedback loop that sustains IRF-4 expression under strong TCR/CD28 stimulation (14). CD30 blockade disrupts this loop by preventing CD30–CD30L engagement and p52/RelB activation. Under strong TCR co-stimulation, TCR-driven canonical NF-κB (p50/p65) alone cannot fully compensate for IRF-4 induction, resulting in IRF-4 reduction (**Supplementary Figure 3**). The situation is even more complex since by stimulation exclusively through the CAR, as it is expected in the therapeutically relevant scenario, IRF-4 is found at different levels dependent on the T-cell donor (**Figure 4D**). In three out of five donors, IRF-4 levels are reduced whereas two show an increase in IRF-4.

We also revealed a moderate increase in pSTAT3 (**Figure 4E**), which likely compensates for the NF-κB loss, as STAT3 and NF-κB regulate overlapping effector genes. pSTAT3 directly binds to the Gzmb promoter driving granzyme B production in T cells (35), relieving CD30-mediated suppression and promoting intracellular granzyme B (**Figure 4F**) and IFN-γ accumulation (**Figure 4G**). This goes along with reduced IL-4, IL-5, and IL-9 release, a hallmark of dampened Th2 polarization, as demonstrated by a Luminex assay (**Figure 4H**). IL-9 reduction was further independently confirmed via ELISA (**Supplementary Figure 4**).

### CD30/CD19 CAR T cells show superior killing of primary CD19^+^ leukemia blasts and in a leukemia xenograft model

**Figure 5:**
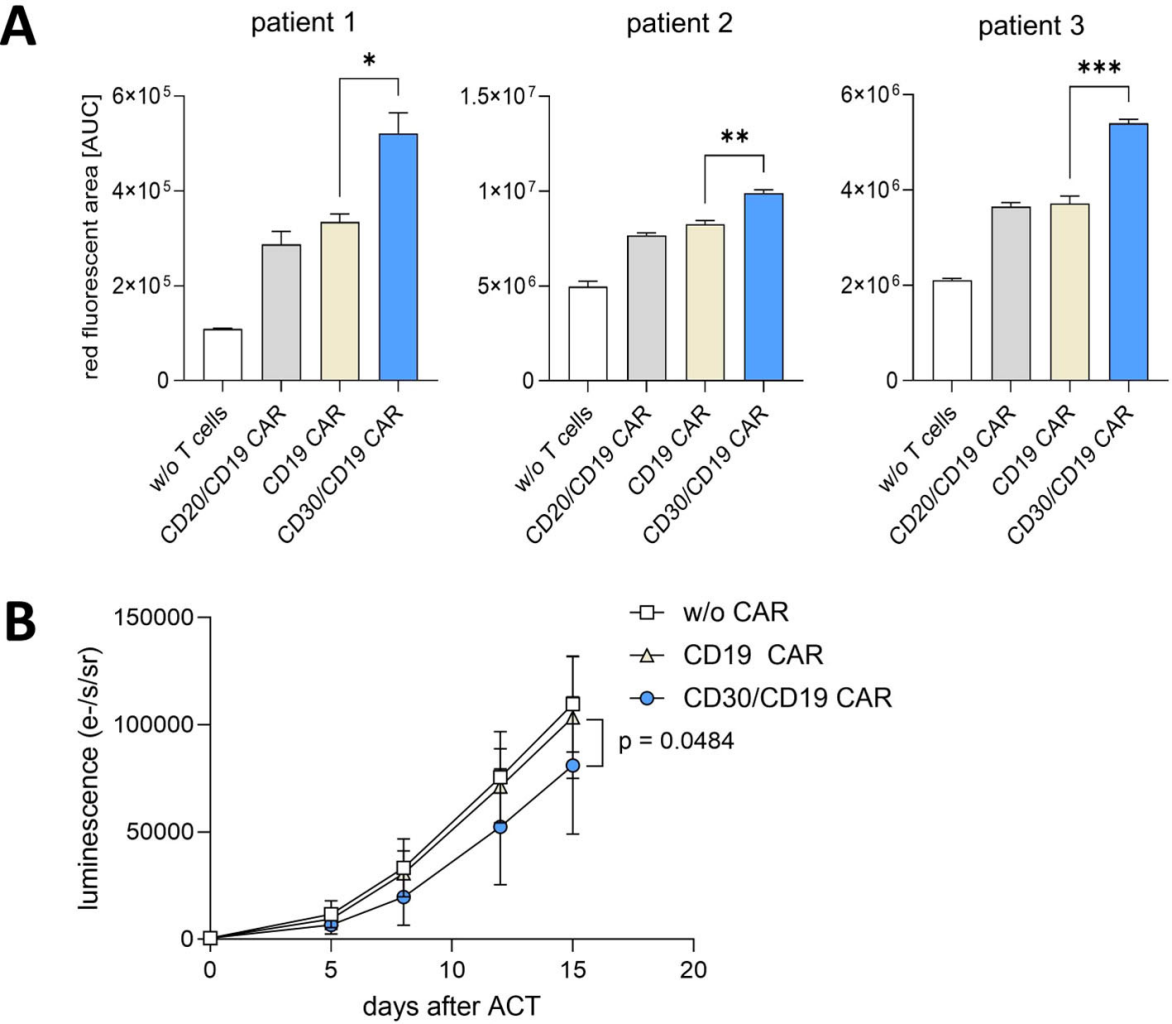
Superior elimination of primary CD19^+^ leukemia blasts and anti-leukemia response. **(A)** CAR T cells were analyzed for killing of primary leukemia blasts from three individual patients diagnosed with B-cell acute lymphoblastic leukemia (B-ALL). Leukemia cells (8 × 10^4^ cells/well) were co-incubated in triplicates with T cells from one out of three healthy donors engineered with the indicated CAR (2 × 10^4^ cells/well each). For control, no T cells were added to leukemia cells. IncuCyte data show area under the curve (AUC) values pooled from technical triplicates of killing assays with the three B-ALL samples + SEM after 12 h of incubation. Statistical analysis was performed using the Student’s t-test: patient 1: p = 0.0159; patient 2: p = 0.0033; patient 3: p = 0.0007. **(B)** NOG mice were intravenously (i.v.) injected with luciferase-labeled CD19^+^ Jeko-1 cells (10^6^ cells). On day 7 (i.e., day 0 of adoptive cell therapy ACT), mice received one i.v. dose of T cells without CAR (10^6^, n=5, transparent symbols), or CD19 CAR T cells (10^6^, n=5, beige symbols), or CD30/19 CAR T cells (10^6^, n=5, blue symbols). Tumor growth was monitored twice per week by luminescence intensity recording (photons/s/sr); graphs represent mean values ± SD. Linear regression analysis over the entire period for CD19 CAR versus CD30/CD19 CAR T cells revealed p=0.0484. *p ≤ 0.05; **p ≤ 0.01; ***p ≤ 0.001.

We addressed whether CD19^+^ cells from leukemia patients were also eliminated with superior efficiency by CD30/CD19 CAR T cells. CD19^+^ primary leukemia blasts from three patients with B-cell acute lymphoblastic leukemia (B-ALL) (**Figure 5**, **Supplementary Figure 5**) were incubated with T cells engineered with the CD30/CD19 CAR, the canonical CD19 CAR, and the CD20/CD19 CAR for comparison, respectively. Again, CD30/CD19 CAR T cells were superior to CD19 CAR T cells in eliminating leukemia cells from each patient as revealed by real-time imaging (**Figure 5A**). Bispecific CD20/CD19 CAR T-cell targeting did not provide a benefit over the canonical CD19 CAR T cells and showed equal cytolytic capacities as the CD19 CAR T cells. The observation holds for all patients’ leukemia samples tested.

To test for activity *in vivo*, luciferase-labeled CD19^+^ Jeko-1 cells were systemically engrafted in NOG mice and CAR engineered T cells injected after 7 days. In this aggressive model and under conditions CD19 CAR T cells did not control leukemic infiltration, CD30/CD19 CAR T cells showed some capacities for slowing down leukemic progress (p=0.0484, linear regression analysis) (**Figure 5B**, **Supplementary Figure 6**), implying some benefits of the CD30/CD19 CAR versus CD19 CAR T cells in controlling leukemic infiltration.

## Discussion

Most patients with B-cell malignancies are suffering from relapses after CAR T-cell treatment, which is thought to be due to the shortcoming in maintaining T cell activation over time. To prevent entry into a dysfunctional state, we here interfered with a process that physiologically orchestrates T-cell activation. Once activated through TCR/CD28 and IL-2, T cells increase CD30L levels on the surface followed by CD30 that binds CD30L and back signals to limit the secondary T-cell response (36, 37). Since CD30 is crucial in this process, we proposed to interfere with the CD30–CD30L interaction by a CAR that contains a CD30 blocking antibody along with a cancer cell targeting domain. The strategy is herein applied to augment T-cell activities against B-cell malignancies by using a CAR that blocks CD30 by the HRS3 scFv and targets B cells by the anti-CD19 scFv FMC63. Noteworthily, CD30/CD19 CAR T cells exhibited improved killing capacities toward CD19^+^CD30^-^ target cells compared with the canonical CD19 CAR T cells *in vitro*. The superior killing capacities by CD30/CD19 CAR T cells further became visible when targeting patient-derived CD19^+^ B-ALL cells, even in a short-term real-time imaging assay. This demonstrates the validity of the observation beyond *in vitro* adapted cell lines. In NOG mice, CD30/CD19 CAR T cells showed modest but statistically significant tumor growth inhibition (p = 0.0484). Testing anti-leukemia activity of CD30/CD19 CAR T cells in a xenograft model using NOG mice, in contrast, is less informative since only long-term adapted, aggressive leukemia cell lines like Jeko-1 establish systemically in these mice whereas human T cells do not home properly to the hematopoietic niche and do not appropriately persist. Moreover, there is less to no cross-reactivity of the CD30 CAR targeting domain with murine CD30 and no murine CD30L binding to human CD30 on T cells, which overall limits monitoring of the CD30 blocking effect on CAR T-cell activities. Future studies using complementary *in vivo* systems (e.g., humanized mice) will further validate and enhance therapeutic translation.

The CD30 targeting domain is required for augmenting the killing capacities since replacing CD30 scFv by the CD20 scFv in the same CAR position did not improve Nalm6 killing compared with CD19 CAR T cells; this is the case although Nalm6 cells express both CD19 and CD20. Consequently, the superiority of the CD30/CD19 CAR is mediated through CD30 targeting and is not due to the bispecific engagement of any two targets. CD30 blocking impacts the T-cell intrinsic functionality whereas bispecific targeting of two antigens on the target cell by the CD20/CD19 CAR provides CAR activation through Boolean “OR” gating of targeted antigens and mediates T-cell activation to a similar degree as upon engagement of only one antigen.

Noteworthily, the position of the respective scFv domains within the CAR extracellular chain is crucial. While the CD30/CD19 CAR improved T-cell functionality, the CD19/CD30 CAR did not compared with the canonical CD19 CAR, pointing to a preferred spatial arrangement of the individual scFv to its cognate protein. Structural modeling of the bidirectional interaction of the CAR with CD19 on the target cell and with CD30 on the CAR T cell sustained our conclusion. Binding the CD19 scFv in the membrane proximal CAR position pulls up a close membrane-to-membrane contact in the optimal range of 14–27 nm for inducing a productive CAR T-cell activation as does the canonical CD19 CAR; the CAR terminal HRS3 scFv, while binding to CD30, does not interfere with the CD19-mediated cell–cell contact. In contrast, the CD19 scFv in the terminal CAR position upon binding allows a broad range of membrane-to-membrane distances of up to 45 nm producing a less stable and less suitable synapse for productive CAR signaling. In general, predictive modeling of multiprotein complexes is limited by the structural complexity, dynamic interactions, flexibility in conformation, and scarce high-resolution data, all challenging the accurate prediction of protein interfaces. The predicted FMC63–CD19 interaction, however, matches the available crystal structure (PDB 7URV) while filling in unresolved flexible loops of CD19. Previous research has shown that HRS3 has a similar binding epitope as the HRS4 mAb, which prevents CD30L binding to CD30 (23, 38). As CD30L binds to the distal region of CD30 (39), the predicted interaction of HRS3 with the distal region of CD30 is plausible. Nevertheless, we see in the structure prediction approach a working hypothesis to visualize our experimental findings as it stands today.

In the presence of CD30/CD19 CAR T cells, the CD30 level on activated T cells decreased compared with canonical CD19 CAR T cells. Fratricide seems to be unlikely the cause since the proportion of Annexin V^+^ cells did not increase under these conditions. Moreover, PI-9, the granzyme-inactivating enzyme, was increased in activated CD30^+^ T cells preventing execution of apoptosis. The observed downregulation of CD30 levels on T cells is likely due to internalization upon CAR binding.

We assume that CAR signaling delivers a T-cell activation signal at donor-dependent levels, which may be sufficient or not to robustly engage either the canonical or non-canonical NF-κB pathways or the CD30-IRF-4 feedback loop. IRF-4 levels were different across T-cell donors upon stimulation with CD19+ HEK cells, likely reflecting differences in the individual T-cell activation thresholds. We conclude that CD30 blockade is predominantly mediated through NF-κB/STAT3 augmenting effector T-cell capacities, independently of IRF-4 modulation during CAR signaling. Taken together, CD30 blockade enhances CD19 CAR T-cell cytotoxic capacities by acting as a negative feedback regulator of effector T-cell function, shifting CD8+ CAR T cells from an immunosuppressive to a more cytotoxic phenotype.

Our study adds to the growing interest in CD30 as a regulatory element in orchestrating the secondary immune response. The interest in CD30 stems from the fact that repetitive CD30 signaling promotes Th2 cell maturation (40) that can be prevented by soluble CD30 or by a CD30L blocking antibody (30), both preventing CD30 downstream signaling. Polarization of CAR T cells toward Th2 cells occurs upon CAR engagement of antigen, which finally results in the decline of T-cell antitumor activities. Secondary CD30 stimulation also increased the number of CD30^+^ cells in the presence of resting allogeneic or autologous T cells (15), again pointing to CD30 as an acute response regulator. As a promoter for a Th2 response, CD30–CD30L stimulation will likely contribute during this process which counteracts a productive Th1 CAR T-cell response. CD30L^+^ cancer cells can also feed into the CD30–CD30L interaction, turning T cells into suppressors of the antitumor response. For instance, AML blasts can express CD30L and upregulate CD30 on T cells thereby reducing their cytolytic effector molecules (19), repressing T-cell proliferation (18) and inducing IL-4 secretion (17, 40). In the context of the interaction of cancer cells with T cells, blocking CD30L binding and diminishing CD30 accessibility by the CD30 CAR will prevent repression and sustain the cytolytic activities of CAR-triggered T cells. In line with these models, interfering with the CD30–CD30L axis through the CD30/CD19 CAR while targeting CD19^+^ leukemia/lymphoma cells is particularly suitable to produce a more persistent pro-inflammatory response than the canonical CD19 CAR.

While dual CD30/CD19 CAR T designs may carry potential off-target risks including autoimmunity, given CD30 expression on activated B and T cells, CD30 CAR T cells show promising efficacy in relapsed/refractory Hodgkin lymphoma or anaplastic large cell lymphoma with no dose-limiting toxicities; grade ≥3 hematologic adverse events were manageable and consistent with lymphodepletion, with no increased infections vs. CD19 CAR T (mild viral increase but fewer severe bacterial cases) (41–43). Integrating dual CD30/CD19 targeting into approved CD19 CAR T manufacturing (e.g. axi-cel, tisa-cel) is feasible via modular adaptations, leveraging established processes for T-cell isolation, activation, transduction, expansion, and cryopreservation.

## Data Availability

All relevant data is contained within the article: The original contributions presented in the study are included in the article/**Supplementary Material**, further inquiries can be directed to the corresponding author/s.

## Supplementary material

Supplementary Figure 1 Surface expression of CD19, CD20, and CD30 on engineered HEK293 and Nalm6 cells. **(A)** HEK293 cells were engineered with CD19, CD30 or both CD19 and CD30 and analyzed by flow cytometry. **(B)** Nalm6 leukemia cells were stained for CD19, CD20 and CD30 surface expression and recorded by flow cytometry. Unstained cells served as control (gray filled line). Median fluorescence intensity (MFI) values of antigen staining are indicated.

Supplementary Figure 2 IncuCyte real-time imaging. Representative images (10x magnification) of cocultured eGFP-positive NALM6 cells together with T cells, CEA CAR T cells, CD19 CAR T cells or CD30/CD19 CAR T cells are shown at three different time points.

Supplementary Figure 3 CD30/CD19 CAR T cells show reduced increase in IRF-4 levels after activation via antiCD3 antiCD28. **(A)** T cells engineered with indicated CAR, and T cells without (w/o) CAR were co-incubated with CD19^-^CD30^-^ HEK293 cells, CD19^+^CD30^-^ HEK cells and with stimulating anti-human CD3 (OKT3) and anti-human CD28 (15E8) antibodies. Intracellular IRF-4 expression levels were recorded by flow cytometry every 12 h for the course of 48 h using a PE-labeled IRF-4 antibody. Data represent mean MFI values of IRF-4 staining pooled from four individual T cell donors + SEM after 24 h of stimulation. Basically, the same data were obtained during the entire course of 48 h incubation (data not shown). Statistical analysis was performed by using Student’s t test; p < 0.05 was considered significant (*p < 0.05, **p < 0.01). **(B)** Representative histograms of IRF-4 expression in anti-CD3/CD28 stimulated CD4+ and CD8+ T cells engineered with the CD19 CAR or the CD30/CD19 CAR.

Supplementary Figure 4 Additional Luminex data, IL-9 ELISA. T cells engineered with indicated CAR (5 x 10^4^ cells/well each) were co-incubated with CD19^+^CD30^-^ HEK (10 x 10^4^ cells/well each) for 48h. **(A)** Luminex data represent mean values of technical triplicates of three individual T cell donors. **(B)** IL-9 in the culture supernatants was recorded by ELISA. Data represent mean values of three technical replicates + SEM of five donors (donor A-E, same as in **Figure 4**). Data was analyzed by unpaired t-test. *p<0.05.

Supplementary Figure 5 Surface expression of CD19, CD20, and CD30 on primary B cell blasts. B cell acute lymphoblastic leukemia (B-ALL) samples from 3 patients were analyzed by flow cytometry for surface expression of CD19, CD20 and CD30. Unstained cells served as control (gray filled line). Median fluorescence intensity (MFI) values of antigen staining are indicated.

Supplementary Figure 6 BLI images. Tumor growth was monitored by luminescence intensity recording (photons/s/cm^2^/sr).
