# Supplementary figures and images for: Blocking CD30 on CD19 CAR T cells augments their functional capacities against B-cell leukemia/lymphoma
Source: Front Immunol. 2026 Apr 30;17:1725641. doi: 10.3389/fimmu.2026.1725641 (PMC13171528; doi:10.3389/fimmu.2026.1725641)

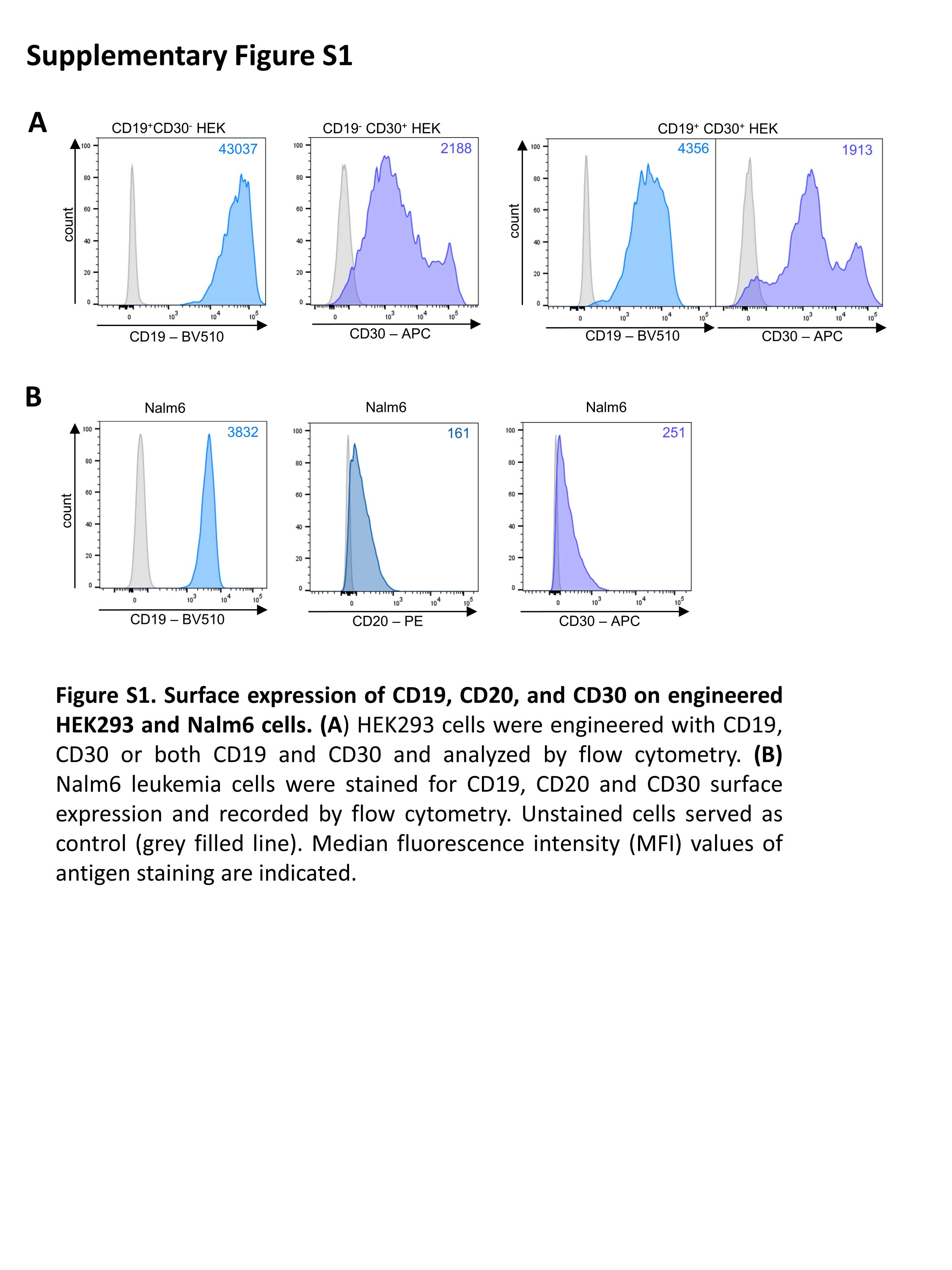

Supplement: Supplementary Figure 1 — Surface expression of CD19, CD20, and CD30 on engineered HEK293 and Nalm6 cells. (A) HEK293 cells were engineered with CD19, CD30 or both CD19 and CD30 and analyzed by flow cytometry. (B) Nalm6 leukemia cells were stained for CD19, CD20 and CD30 surface expression and recorded by flow cytometry. Unstained cells served as control (gray filled line). Median fluorescence intensity (MFI) values of antigen staining are indicated. [file Image1.jpeg]

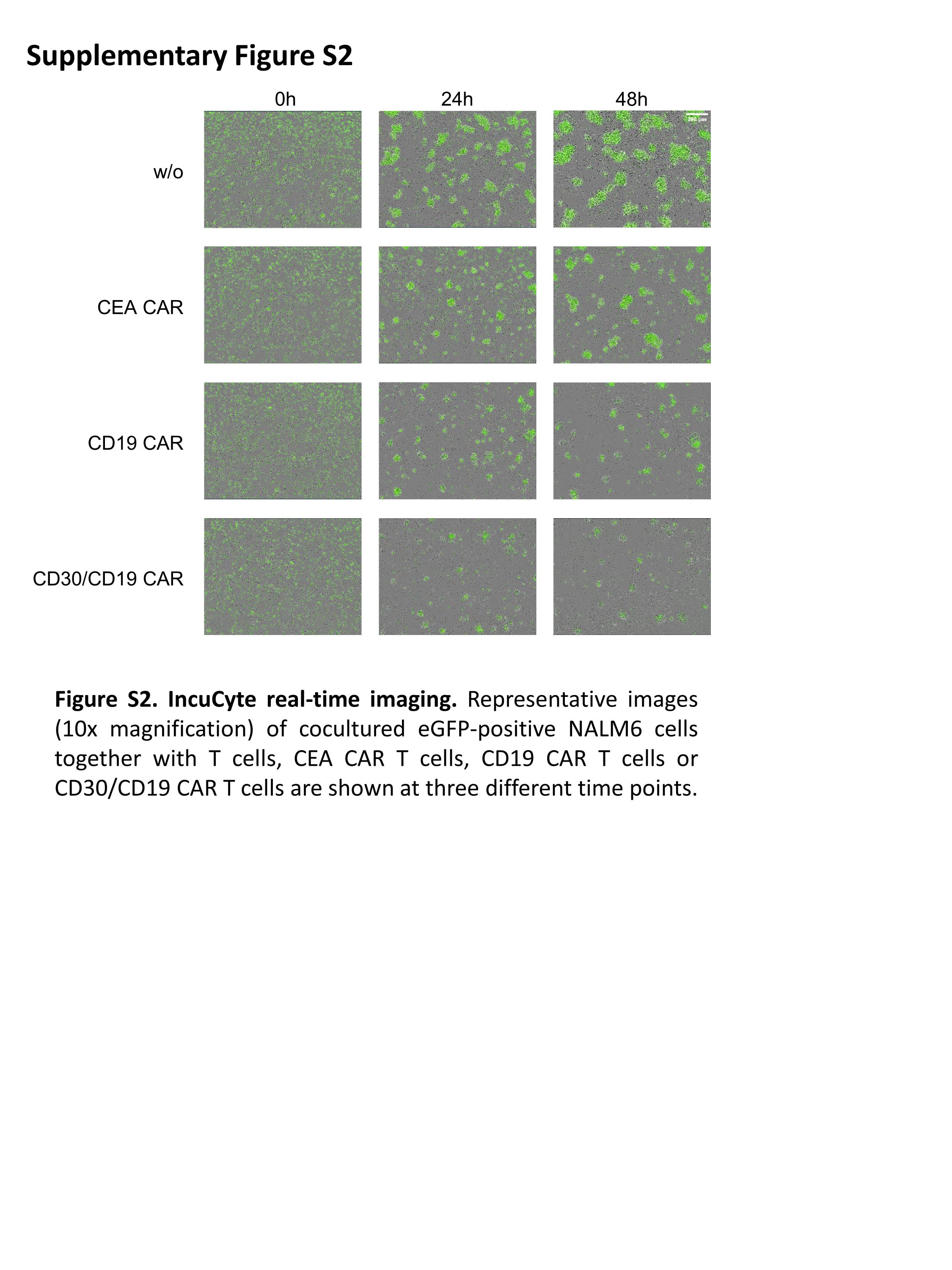

Supplement: Supplementary Figure 2 — IncuCyte real-time imaging. Representative images (10x magnification) of cocultured eGFP-positive NALM6 cells together with T cells, CEA CAR T cells, CD19 CAR T cells or CD30/CD19 CAR T cells are shown at three different time points. [file Image2.jpeg]

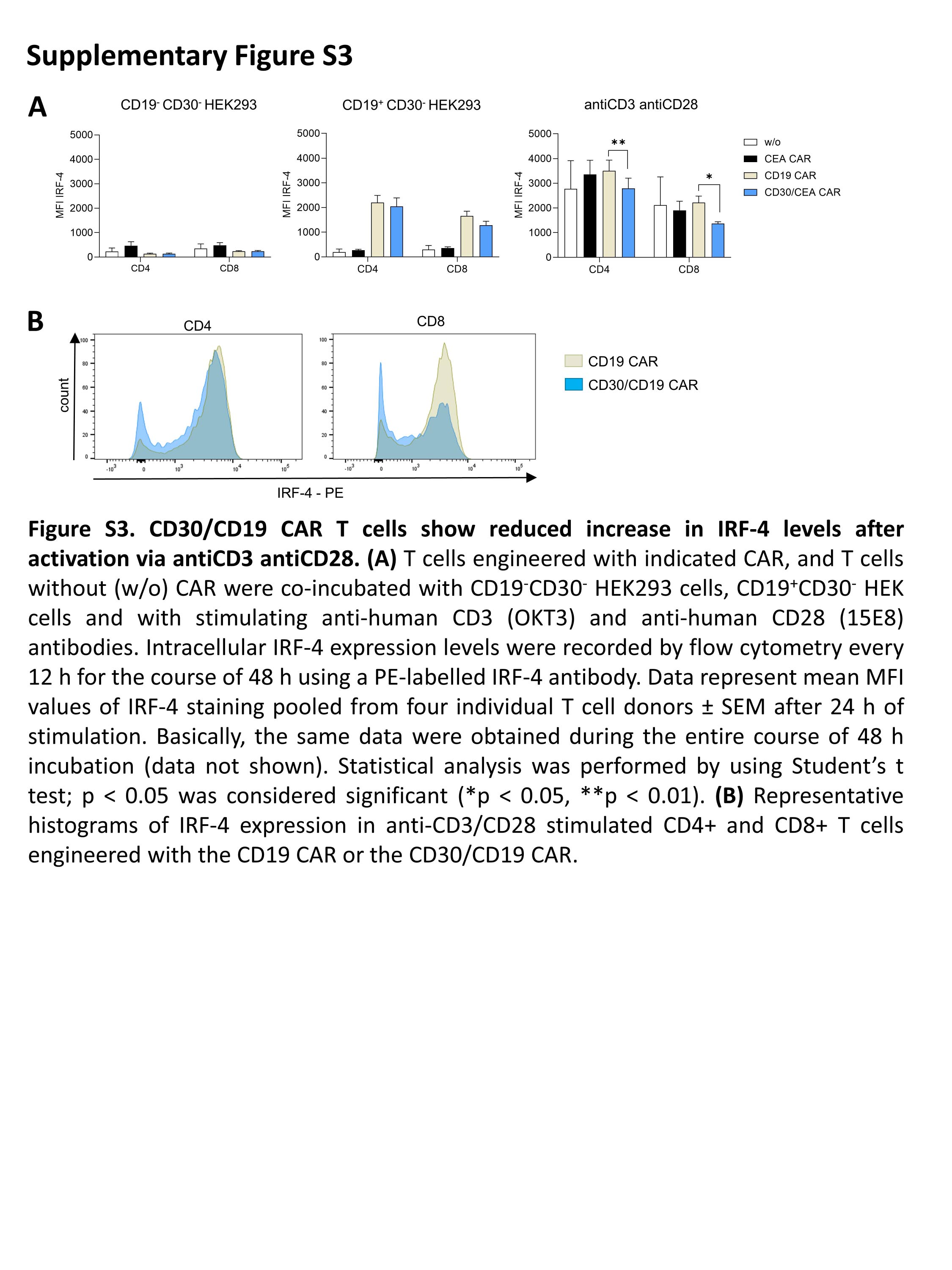

Supplement: Supplementary Figure 3 — CD30/CD19 CAR T cells show reduced increase in IRF-4 levels after activation via antiCD3 antiCD28. (A) T cells engineered with indicated CAR, and T cells without (w/o) CAR were co-incubated with CD19-CD30- HEK293 cells, CD19+CD30- HEK cells and with stimulating anti-human CD3 (OKT3) and anti-human CD28 (15E8) antibodies. Intracellular IRF-4 expression levels were recorded by flow cytometry every 12 h for the course of 48 h using a PE-labeled IRF-4 antibody. Data represent mean MFI values of IRF-4 staining pooled from four individual T cell donors + SEM after 24 h of stimulation. Basically, the same data were obtained during the entire course of 48 h incubation (data not shown). Statistical analysis was performed by using Student’s t test; p < 0.05 was considered significant (*p < 0.05, **p < 0.01). (B) Representative histograms of IRF-4 expression in anti-CD3/CD28 stimulated CD4+ and CD8+ T cells engineered with the CD19 CAR or the CD30/CD19 CAR. [file Image3.jpeg]

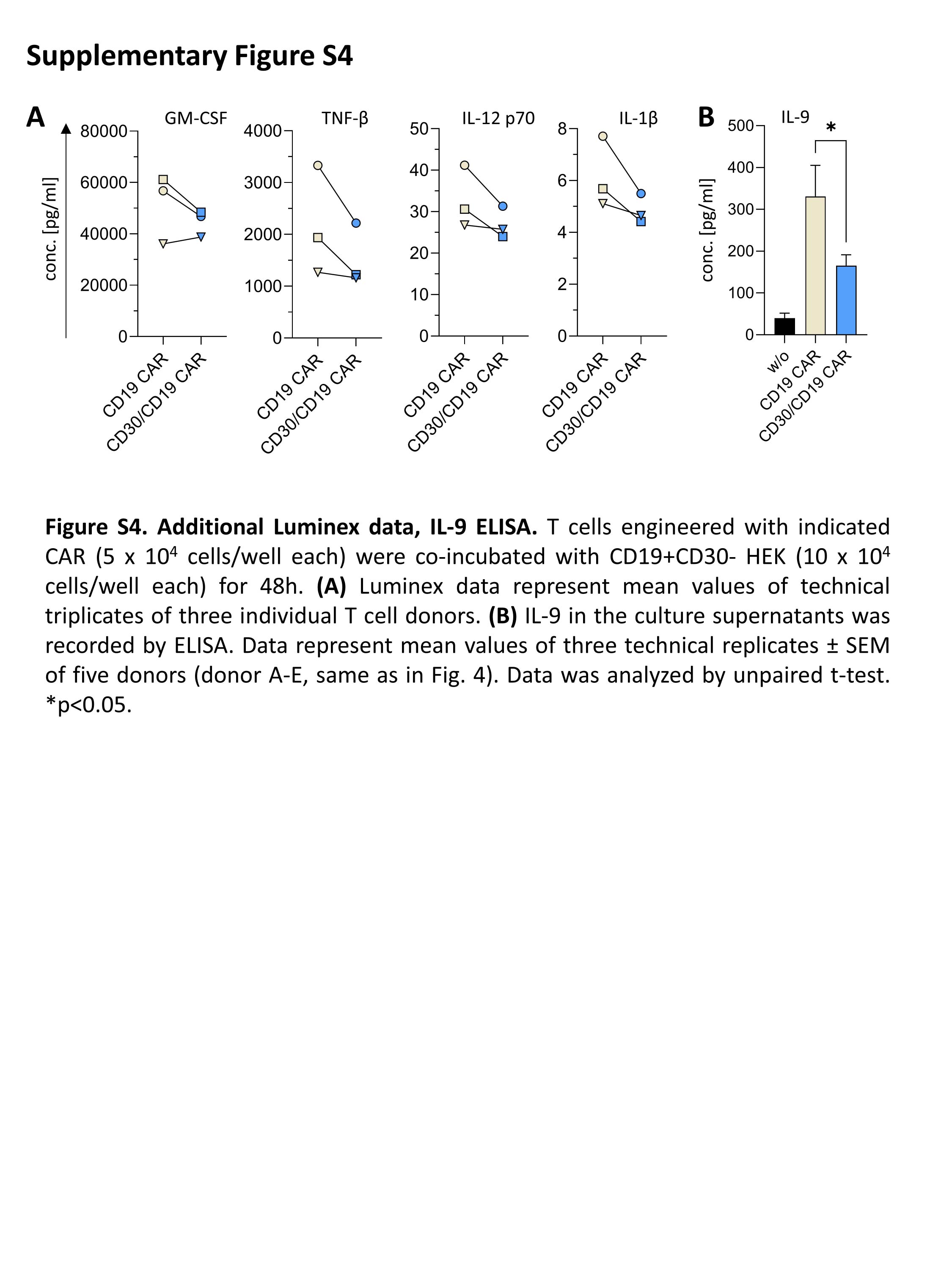

Supplement: Supplementary Figure 4 — Additional Luminex data, IL-9 ELISA. T cells engineered with indicated CAR (5 x 10^4^ cells/well each) were co-incubated with CD19+CD30- HEK (10 x 104 cells/well each) for 48h. (A) Luminex data represent mean values of technical triplicates of three individual T cell donors. (B) IL-9 in the culture supernatants was recorded by ELISA. Data represent mean values of three technical replicates + SEM of five donors (donor A-E, same as in Figure 4). Data was analyzed by unpaired t-test. *p<0.05. [file Image4.jpeg]

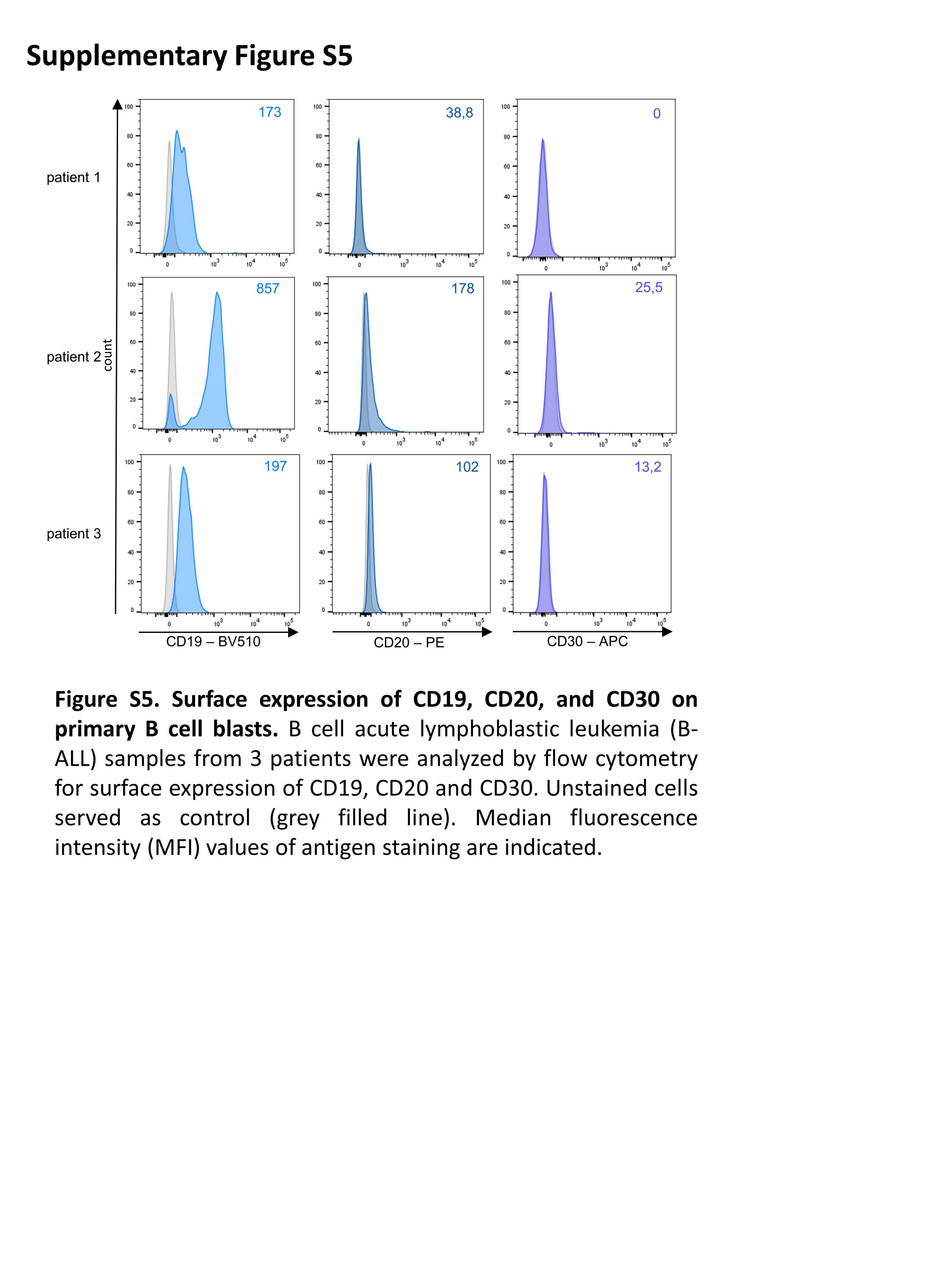

Supplement: Supplementary Figure 5 — Surface expression of CD19, CD20, and CD30 on primary B cell blasts. B cell acute lymphoblastic leukemia (B-ALL) samples from 3 patients were analyzed by flow cytometry for surface expression of CD19, CD20 and CD30. Unstained cells served as control (gray filled line). Median fluorescence intensity (MFI) values of antigen staining are indicated. [file Image5.jpeg]

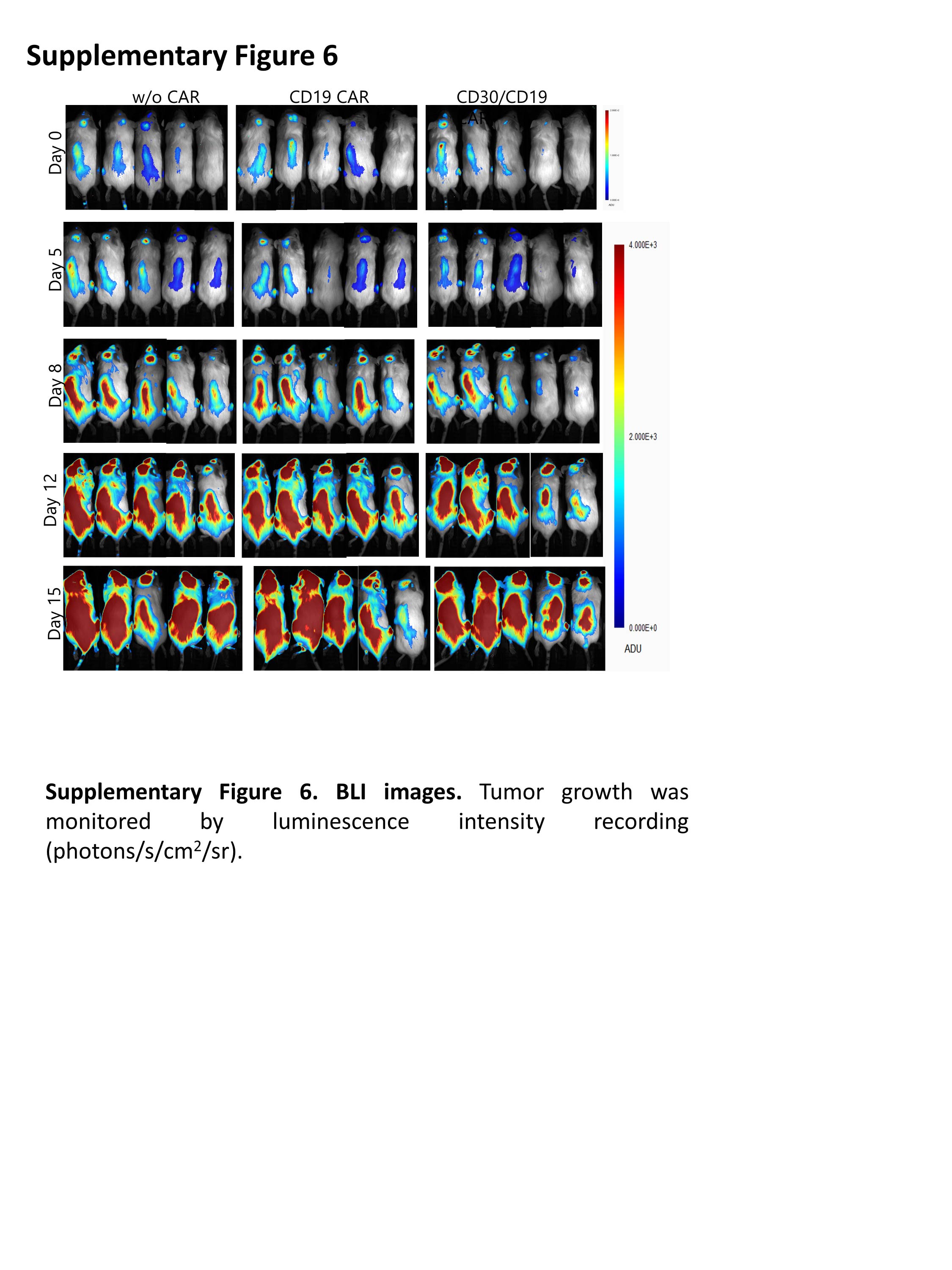

Supplement: Supplementary Figure 6 — BLI images. Tumor growth was monitored by luminescence intensity recording (photons/s/cm2/sr). [file Image6.jpeg]
